# Perinatal Outcome of Babies Delivered to Eclamptic Mothers: A Prospective Study from a Nigerian Tertiary Hospital

**Published:** 2009-12

**Authors:** Innocent O. George, Israel Jeremiah

**Affiliations:** 1 *Departments of Paediatrics, University of Port-Harcourt Teaching Hospital, Port-Harcourt, Nigeria*; 2 *Department of Obstetrics and Gynaecology, University of Port-Harcourt Teaching Hospital, Port-Harcourt, Nigeria*

**Keywords:** perinatal outcome, eclampsia, antenatal care

## Abstract

Background: Eclampsia is a leading cause of maternal and perinatal mortality in Nigeria. Preventive and interventional measures have been shown to reduce maternal mortality and morbidity with no significant beneficial effect on neonatal outcomes. The aim of this study is to assess the perinatal outcome of eclampsia at the University of Port-Harcourt Teaching Hospital (UPTH). Materials and methods: This prospective, cross-sectional study was conducted on 88 consecutive patients presenting with antepartum eclampsia at the UPTH between 1^st^ January 2007 and 31^st^ December 2008. A protocol was developed and used to collect information about socio-demographic characteristics, mode of delivery, perinatal complications and outcome. Data collected was entered into a spread sheet using SPSS 15.0 for Windows^®^ statistical software which was also used for analysis. Chi square was used to test relationship between variables. P value<0.05 was considered statistically significant. Results: There were a total of 5488 deliveries at the University of Port-Harcourt Teaching Hospital from January 2007 to December 2008. Of these, 5,310 were live births while 404 were perinatal deaths giving perinatal mortality rate of 73.6 per 1000 live births. Eighty eight (1.6%) of the mothers were eclamptic. Eight (9.1%) were booked, 80 (90.1%) were unbooked. The mean gestational age at delivery was 35.1 ± 4.2 weeks. The main mode of delivery was by emergency caesarian section in 49 (55.7%) of the patients. Others were spontaneous vertex delivery (34.1%), assisted vaginal breech delivery (6.8%) and instrumental vaginal delivery (3.4%). Fifty four babies (61.4%) were admitted into the Special Care Baby Unit. Indications for admission include prematurity (n=23), low birth weight (n=10), severe birth asphyxia (n=12), neonatal jaundice (n=4) and neonatal sepsis (n=5). There were 37 perinatal deaths giving a perinatal mortality rate of 411 per 1000 live births. Of the mortalities, 19 were intrauterine foetal deaths, while 18 were early neonatal deaths. Causes of death include severe birth asphyxia (n=6), respiratory distress syndrome (n=4), prematurity (n=4), neonatal jaundice (n=1) and sepsis (n=3). Conclusion: Eclampsia is a major contributor of perinatal mortality and morbidity in Nigeria. Detection and appropriate management of preeclampsia is critical to reduce the risk of eclampsia.

## INTRODUCTION

Perinatal mortality is an important indicator of the status of maternal and child health, the conditions of obstetric care and the level of economic development of a community (1). It includes stillborn babies (SB) of more than 28 weeks of gestation and deaths occurring within the first week of life (early neonatal deaths) (1). The perinatal mortality rate (PMR) reflects both the characteristics of reproductive health and the quality of antenatal care, delivery, and newborn care (2). At the global level, an estimated 7.5 million perinatal deaths take place each year, most of which are in developing countries (3, 4). The perinatal mortality vary between different regions for instance, it ranges from less than 10 per 1,000 in most developed countries to up to 60 per 1,000 in certain regions of Asia and Africa (3, 4).

Eclampsia is defined as the occurrence of fit or seizure in a patient with signs and symptoms of pre-eclampsia in the absence of underlying neurologic disease (5). It is known to contribute significantly to maternal and perinatal mortality in developing countries like Nigeria (6). It is, however, a rarity in developed countries. For example in the United States, an incidence of 4.3/10000 was reported while 4.9/10000 was reported in England (7, 8). This is not the case with developing nations where the incidence is much higher (6). The incidences increases with deprivation and lack of maternal healthcare organization (9). For instance, the incidences reported in a study conducted in Nigeria and South-Africa are 42/10000 and 36/10000 respectively (10, 11). A ten year study in a rural hospital in Benin, Nigeria gave a case fatality rate of 15% and a perinatal mortality rate of 195 per 1000 births (12). Several institutions in Nigeria have reported maternity mortality rates of 330 per 100,000 contributing 21.1% of the maternal deaths and a perinatal mortality of 16.3% (13). The high rate of complications in developing countries has been attributed to lack of antenatal and intrapartum care for many of the obstetric population.

The pathophysiology attributed to this condition is as seen in pre-eclampsia. Virtually every organ is affected in the woman. Risk factors to the fetus and eventual perinatal death are intrauterine growth restrictions, low birth weight and prematurity as result of placental insufficiency and chronic hypoxia (due to the underlying pre-eclampsia rather than the eclampsia itself). Other risk factors are sepsis, birth asphyxia and its sequalae, and abruptio placenta.

There are few published data on perinatal mortality as a result of eclampsia. This study, therefore, was done to determine the perinatal outcome following eclampsia at the University of Port-Harcourt Teaching Hospital (UPTH), Port-Harcourt. Vital statistics obtained from this study may serve an important source of information to guide the public health policy makers and health care providers.

## MATERIALS AND METHODS

This prospective, cross-sectional study was conducted on 88 consecutive patients presenting with ante partum eclampsia at the University of Port Harcourt Teaching Hospital between 1^st^ January 2007 and 31^st^ December 2008.

Diagnosis of eclampsia was based on diastolic blood pressure measurement greater than 90mmHg, proteinuria and convulsion (5).

All perinatal deaths including stillbirths (SBs) and early neonatal deaths (ENNDs) within 0–7 days of birth were included and pregnancies less than 24 weeks were excluded from this study.

A protocol was developed and used to collect information about socio-demographic characteristics, mode of delivery, perinatal complications and outcome. Data collected was entered into a spread sheet using SPSS 15.0 for Windows^®^ statistical software which was also used for analysis. Chi square was used to test relationship between variables. P value <0.05 was considered statistically significant.

## RESULTS

The total number of deliveries at UPTH from January, 2007 to December, 2008 was 5488, 5310 of these were live babies while 404 were perinatal deaths giving a perinatal mortality rate of 73.6 per 1000 live births.

Eighty eight women presented with eclampsia over the study period resulting in an incidence of eclampsia of 16 per 1000 deliveries (1.6%). Unbooked patients who had received inadequate or no antenatal care comprised 90.9% of the women who presented at our hospital with eclampsia (Table 1).

**Table 1:** The effect of antenatal care, maternal age, parity and Plurality on Perinatal Mortality Rate

Risk factor	Stillbirth			Early neonatal death			PMR/1000
	Total births	Stillbirths (%)	P-value	Live births	ENND (%)	P-value	

Antenatal care							
Booked	8	0 (0.0)	<0.001	8	0 (0)	<0.001	0.0
Unbooked	80	19 (23.8)		61	18 (42.9)		411.1
Maternal age (years)							
16 – 19	9	2 (22.2)		7	2 (28.6)		44.4
20 – 24	24	8 (33.3)		16	5 (31.3)		144.4
25 – 29	29	3 (10.3)		26	5 (19.2)		88.9
30 – 34	15	3 (20.0)		12	3 (25.0)		66.7
35 – 39	6	1 (16.7)		5	2 (40.0)		33.3
≥ 40	5	2 (40.0)		3	1 (33.3)		33.3
≤34	77	16 (20.8)	0.88	61	15 (24.6)	0.61	344.4
≥35	16	3 (18.8)		13	3 (23.0)		66.7
Maternal Parity							
0 – 4	78	16 (20.5)	0.69	62	15 (24.2)	0.58	344.4
≥ 5	10	3 (30.0)		7	3 (42.9)		66.7
Plurality							
Singleton	86	19 (22.1)		67	18 (26.9)		411.1
Multiple	4	0 (0.0)		4	0 (0.0)		0.0

ENND, Early neonatal death; PMR, Perinatal mortality rate.

The mean gestational age at presentation was 35.04 ± 4.21 weeks with a range of 24 weeks – 43 weeks and 57.1% of them presenting preterm (Table 2).

Caesarean delivery was the commonest mode of delivery 49 (55.7%) among the subjects with eclampsia as shown in table 2.

**Table 2:** The effect of sex, mode of delivery, birth weight and gestational age on Perinatal Mortality Rate

Risk factor	Stillbirth			Early neonatal death			PMR/1000
	Total births	Stillbirths (%)	P-value	Live births	ENND	P-value	

Sex							
Male	45	14 (31.1)	0.06	31	8 (25.8)	0.95	244.4
Female	45	5 (11.1)		40	10 (25.0)		166.7
Mode of delivery							
SVD	30	13 (43.3)		17	8 (47.1)		233.3
Emergency C/S	49	3 (6.1)	0.001	46	8 (17.4)	0.04	122.2
Breech delivery	6	1 (16.7)		5	2 (40.0)		33.3
Instrumental	3	2 (66.7)		1	0 (0.0)		22.2
Birth weight (gram)							
<1000	3	1 (33.3)		2	2 (100)		33.3
1000 – 1499	11	1 (9.1)		10	7 (70.0)		88.9
1500 – 1999	10	2 (20.0)		8	4 (50.0)		66.7
2000 – 2499	17	2 (11.8)		15	1 (6.7)		33.3
2500 – 2999	19	6 (31.6)		13	1 (7.7)		77.8
3000 – 3499	20	5 (25.0)		15	1 (6.7)		66.7
3500 – 3999	7	2 (28.6)		5	1 (20.0)		33.3
≥ 4000	1	0 (0.0)		1	1 (100)		11.1
<2500	41	6 (14.6)		35	14 (40)		222.2
≥2500	49	13 (27.7)		36	4 (11.8)	0.03	188.9
Gestational Age(weeks)							
≤ 28	6	2 (33.3)		4	4 (100)		66.7
29 – 36	26	2 (7.7)	0.15	24	7 (29.2)	0.12	100.0
≥ 37	24	5 (20.8)	0.24	19	2 (10.5)	0.01	77.8
Uncertain	32	10 (31.3)		22	5 (22.7)		166.7

ENND, Early neonatal death; PMR, Perinatal mortality rate.

The total number of births in our series was 90, which included 86 singleton births and 2 sets of twins with a mean birth weight of 2.44 ± 8.18 Kg and a range 0.7 Kg – 4.0 Kg.

Fifty four babies (61.4%) were admitted into the Special Care Baby Unit. The indications for admission were; prematurity (n=23), low birth weight (n=10), severe birth asphyxia (n=12), neonatal jaundice (n=4) and neonatal sepsis (n=5).

There were 37 perinatal deaths, giving a perinatal mortality rate of 411.1 per 1000 live births of babies born to eclamptic mothers. These included 19 still births (51.4%) and 18 early neonatal deaths (48.6%). Birth asphyxia (33.3%), respiratory distress syndrome (22.2%) and prematurity (22.2%) were the commonest causes of neonatal deaths (Table 3).

**Table 3:** Causes of neonatal Deaths

Cause	No. of Pts	Percentage

Birth Asphyxia	6	33.3
Respiratory distress syndrome	4	22.2
Prematurity	4	22.2
Sepsis	3	16.7
Jaundice	1	5.6

Babies of unbooked mothers accounted for 66.7% of the perinatal deaths. This was significantly higher than the perinatal deaths among babies of booked mothers (p<0.001). Perinatal mortality was also significantly higher among babies delivered vaginally than those delivered by caesarean section (p=0.04; OR=2.27 [0.95 – 5.52]). Low birth weight babies had a significantly higher risk of perinatal mortality than normal weight babies p=0.03; OR= 1.41 [0.65 – 3.25])

## DISCUSSION

Eclampsia is a significant cause of maternal and perinatal morbidity and mortality, particularly in developing countries, where the incidence is still high (6). It is reported to affect 0.015% to 0.05% of the pregnant women in the West, compared (13) to an incidence of 1.6% in our series. This figure is lower than an Indian study which reported an incidence of 2.2% (14) but higher than reports from Africa (0.36% to 0.42%) (6, 11). This high incidence we reported in our series is a reflection of our failure to prevent eclamptic convulsions in a substantial number of pregnant women. It has been shown that physician’s error, patient failures and abrupt or late onset of eclampsia have been reported to be responsible for failure to prevent eclampsia (15). In this study, patient’s failure contributed to the development of eclampsia as most patients were unbooked (90.9%) and had received inadequate or no antenatal care.

The perinatal mortality rate (PMR) has been widely used to assess the outcome of late fetal and early neonatal life and is generally regarded as an index of how well society looks after women in their reproductive years. The PMR of 411 per 1000 live birth due to eclampsia in this study is quite high compared with the overall perinatal mortality rate of 73.6 per 1000 live birth. This shows that eclampsia is a major contributor to perinatal mortality.

Low birth weight (LBW) has been shown to be a key determinant of perinatal mortality (6). In this study LBW babies (Birth weight <2.5 Kg) significantly fared worse than their full sized counterparts. This agrees with most studies (16–18).

Birth asphyxia was the commonest cause of perinatal mortality in our study, accounting for one third of the perinatal deaths. This had been documented previously (19) and it has been suggested that perinatal deaths due to asphyxia, rather than crude PMR, be used as a measure of the efficacy of perinatal care (19).

Babies delivered by Caesarean section had a better perinatal outcome than their counterparts delivered vaginally. This would suggest that a more generous use of Caesarean section for delivery especially in the circumstance of a stable maternal condition and a live baby would improve perinatal survival and as such is advocated.

Fifty percent of all the perinatal death in our study were stillbirths. All these stillbirths occurred among the unbooked mothers. Thus, we believe that advances in antenatal care and appropriate timing of delivery in eclamptics will be effective in increasing perinatal survival rates for infants born to these mothers.

## CONCLUSION

Eclampsia remains a major contributor to perinatal mortality in Nigeria. The major avoidable contributing factor is lack or absence of antenatal care. Hence, an improvement in the antenatal care and neonatal care services will be effective in reducing the incidence of eclampsia, as well as the morbidity and mortality associated with it.

## References

[1] Yu V (2003). Global, regional and national perinatal and neonatal mortality. J. Perinat. Med.

[2] Jackson DJ, Lang JM, Ganiats TG (1999). Epidemiological issues in perinatal outcomes research. Paediatr Perinat Epidemiol.

[3] World Health Organization (2006). Neonatal and perinatal mortality: country, regional and global estimates.

[4] Stanton C, Lawn JE, Rahman H, Wilczynska-Ketende K (2006). Stillbirth rates: delivering estimates in 190 countries. Lancet.

[5] Kwawukume EY, Kwawukume EY, Emuveyan EE (2002). Hypertension in Pregnancy. In: Comprehensive Obstetrics in the tropics.

[6] Onwuhafua PI, Oguntayo A (2006). Perinatal mortality associated with eclampsia in Kaduna, Northern Nigeria. Nigerian Journal of Medicine.

[7] Sharara HA, Othman SY (2002). A review of eclampsia in Qatar. Int. J. Gynecol. Obstet.

[8] Jamelle RN (1997). Eclampsia- a taxing situation in the Third World. Int. J. Gynecol. Obstet.

[9] Bergstrom S, Lawson JB, Harrison KA, Bergstrom S (2001). Pre-eclampsia and Eclampsia. In: Maternity Care in Developing Countries.

[10] Onwuhafua PI, Onwuhafua A, Adze J, Mairami Z (2001). Eclampsia in Kaduna State of Nigeria: a proposal for a better outcome. Niger J. Med.

[11] Mwinyoglee J, Amoko DH, Simelela N (1996). Eclampsia at Ga-Rankuwa Hospital. S. Afr. J. Med.

[12] Igberase GO, Ebeigbe PN (2006). Eclampsia: ten-years of experience in a rural tertiary hospital in the Niger delta, Nigeria. Journal of Obstetrics and Gynaecology.

[13] Taneer CE, Hakverdi AU, Aban M (1996). Prevalence, management and outcome in eclampsia. Int. J. Gynecol Obstet.

[14] Swain S, Ojah KN, Prakash A (1993). Maternal and perinatal mortality due to eclampsia. Indian Pediatr.

[15] Sibai BM, Abdella TN, Spinnata JA (1981). Eclampsia: observations from 67 recent cases. Obstet. Gynecol.

[16] Shaheen BM, Hassan L, Obaid M (2003). Eclampsia, a major cause of maternal and perinatal mortality: a prospective analysis at a tertiary care hospital of Peshawar. J. Pak. Med. Assoc.

[17] Kenyon PJI (1991). Perinatal mortality statistics in Harare 1980–1989. Centr. Afr. J. Med.

[18] Kusin JA, Kardjati S, de With C (1989). Infant mortality in Mandura, Indonesia. Implication for action. J. Trop. Pediatr.

[19] Macfarlane A, Chalmers I, Hull D (1981). Problems in the interpretation of perinatal mortality statistics. Recent Advances in Paediatrics (6).

